# Formative evaluation of an employee-driven approach to improve care in the dying phase in hospitals

**DOI:** 10.1017/S1478951525100400

**Published:** 2025-07-18

**Authors:** Sophie Meesters, Sukhvir Kaur, Viola Milke, Christin Herrmann, Aneta Schieferdecker, Nikolas Oubaid, Karin Oechsle, Holger Schulz, Holger Pfaff, Raymond Voltz, Kerstin Kremeike

**Affiliations:** 1 Palliative Medicine, University Hospital Augsburg, Augsburg, Germany; 2 Department of Palliative Medicine, Faculty of Medicine and University Hospital, University of Cologne, Cologne, Germany; 3 Palliative Care Unit, Department of Oncology, Hematology and BMT, University Medical Center Hamburg-Eppendorf, Hamburg, Germany; 4 Chair of Quality Development and Evaluation in Rehabilitation, Institute of Medical Sociology, Health Services Research and Rehabilitation Science, Faculty of Human Sciences & Faculty of Medicine and University Hospital, University of Cologne, Cologne, Germany; 5 Department of Medical Psychology, University Medical Center Hamburg-Eppendorf, Hamburg, Germany; 6 Center for Integrated Oncology Aachen Bonn Cologne Duesseldorf (CIO ABCD), Faculty of Medicine and University Hospital, University of Cologne, Cologne, Germany; 7 Center for Health Services Research (ZVFK), Faculty of Medicine and University Hospital, University of Cologne, Cologne, Germany

**Keywords:** Terminal care, quality of health care, participatory research, hospitals, mixed-methods

## Abstract

**Objectives:**

The hospital setting is often perceived as slow to change. While employee-driven approaches offer a promising alternative to traditional top-down methods, guidance is limited. This study provides a description and formative evaluation of an employee-driven working group (WG) approach to tailor ward-specific measures to improve care in the dying phase. The aim is to evaluate the WG process and offer practical insights for transferability to other hospitals.

**Methods:**

Formative mixed-methods evaluation of a WG process to tailor ward-specific evidence-informed measures on 10 wards outside specialized palliative care at 2 German medical centers. To analyze factors relevant for the WG process, the *Consolidated Framework for Implementation Research 2.0* was applied. Data included baseline evaluation (medical record analysis, staff survey and focus groups, informal caregiver interviews), WG protocols, and an online survey with WG participants.

**Results:**

Multiprofessional WGs were established on all hospital wards, with an average of 7 meetings per ward within 1 year and 4 participants per meeting. Adapting the process to participants’ wishes and needs were crucial, particularly regarding the desired degree of external input. We identified 4 barriers (e.g. declining participation, institutional limits) and 7 facilitators (e.g. involvement of staff in leading positions, multiprofessional composition). The WGs tailored 34 measures, e.g. team meetings to improve communication within the team. Participants’ views were generally positive: 91% felt able to share their thoughts, 66% were satisfied with the outcome, and 77% would participate again.

**Significance of results:**

The employee-driven approach was feasible and useful for tailoring ward-specific measures. However, integrating top-down elements proved to be beneficial. The identified barriers and facilitators provide insights for transferring an employee-driven approach to other hospitals to improve care in the dying phase outside specialized palliative care settings.

**Clinical trial registration:**

The study was registered in the German Clinical Trials Register (DRKS00025405).

## Introduction

Driving change in the hospital setting is challenging. Literature on implementing innovations highlights a change-resistant environment with barriers like lack of time and staff resources, complex care processes or entrenched hierarchies (Cadeddu et al. 2023). Moreover, innovations often follow a top-down approach, facing mistrust and resistance (Burcharth et al. 2014; Cadeddu et al. 2023). While these challenges exist in curative settings, they are as or even more present in the context of care in the dying phase, particularly in hospital wards with a predominantly curative focus (Docherty et al. 2008; Mayland et al. 2017; Robinson et al. 2014). Patients and informal caregivers frequently reported inadequate quality of care in the dying phase in these contexts (Docherty et al. 2008; Mayland et al. 2017; Robinson et al. 2014). In Europe, approximately 50% of people die in hospitals outside specialized palliative care, leading to initiatives like the *Liverpool Care Pathway* (Ellershaw and Wilkinson 2011), the *Best Care of Dying* recommendations (Ellershaw and Lakhani 2013; Montag et al. 2014) or the *German Guideline Palliative Care for Patients with Incurable Cancer* (German Guideline Programme in Oncology 2020). However, these rather complex and inflexible initiatives faced implementation challenges (Di Leo et al. 2015; Koffman et al. 2019).

Employee-driven (ED) approaches offer a promising alternative (Cadeddu et al. 2023). They often involve small, tailored interventions that enable short-term testing of new ideas and flexible adaption (Cadeddu et al. 2023). Challenges include the development of new structures and the question of how to organize and enable ED innovations (Cadeddu et al. 2023). Despite their potential, research that provides insights and guidance on practice and creating conditions of ED approaches is limited (Cadeddu et al. 2023).

Our project “Dying in hospital in Germany – Optimising care in the dying phase” adopts an ED approach using working groups (WGs) to tailor ward-specific evidence-informed measures. The objective is to improve the quality of care for patients in the dying phase on wards that are not specialized in palliative care (Kremeike et al. 2022). The aim of this study is to provide both a comprehensive description and formative evaluation of the ED WG approach to tailor ward-specific measures. We applied the *Consolidated Framework for Implementation Research 2.0* (*CFIR 2.0*) (Damschroder et al. 2022) to understand how such an approach can be structured and what conditions facilitate or hinder its implementation. By detailing the process, the research team aims to generate insights that support replication and adaption to other hospital settings.

## Methods

### Study design

**Figure 1.**
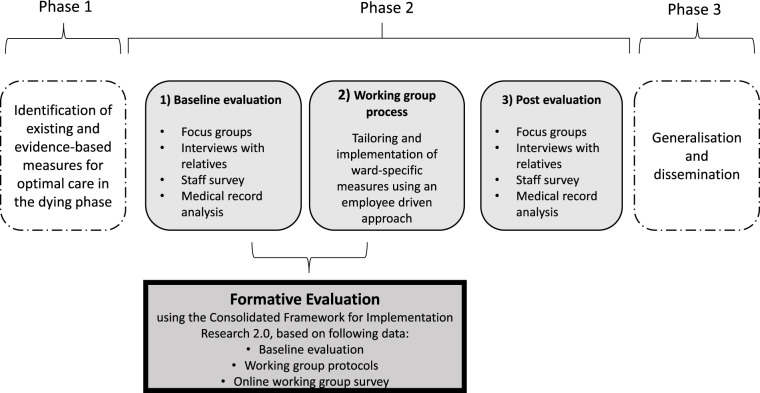
Project phases of the study *Dying in hospital in Germany– Optimising care in the dying phase* (Kremeike et al. 2022)

This mixed-methods formative evaluation was carried out as part of a single-arm baseline-post study including 3 phases (Kremeike et al. 2022). The formative evaluation described below focuses on the tailoring of ward-specific measures in WGs within the second study phase (see Fig. 1).

### Setting and recruitment

In one medical center, invitations were sent to department directors of 14 wards, of which 8 declined and 1 did not respond. Reasons for declining participation varied: 3 wards cited low mortality rates, while 4 wards pointed out to specific challenges and burdens posed by their patient populations, such as dying infants, that do not align with overall study aims. One ward declined due to their involvement in other research projects. In the other medical center, the research team agreed on selecting 2 intensive care and 3 peripheral wards, and contacted wards based on high mortality rates. All wards responded and agreed to participate, requiring the research team to make a final selection of 5 wards. Finally, 6 ICUs and 4 GWs located at 2 German medical centers participated in our study. All wards are not specialized in palliative care but regularly care for dying patients and cover a wide range of disciplines.

### The WG process

We planned to start the WG process on all wards in February 2022 with an introductory meeting, followed by periodic hour-long meetings every 4 to 6 weeks until end of 2022. The WGs should consist of a minimum of 3 to 5 ward staff members of different professions, supported by the research team and a palliative care-trained nurse or physician. The research team was responsible for organizing the meetings (e.g. sending meeting invitations and protocols) and supporting tasks, such as preparing drafts and providing relevant literature. The nurse or physician with training in specialized palliative care from the respective hospital attended the meetings. The purpose of their attendance was to provide expertise, answer questions related to palliative care and ensure the clinical appropriateness of the developed measures. Recruitment was coordinated through ward contacts, typically senior physicians for medical staff and nurse team leaders for nurses, therapists, and counsellors.

### Data collection

**Figure 2.**
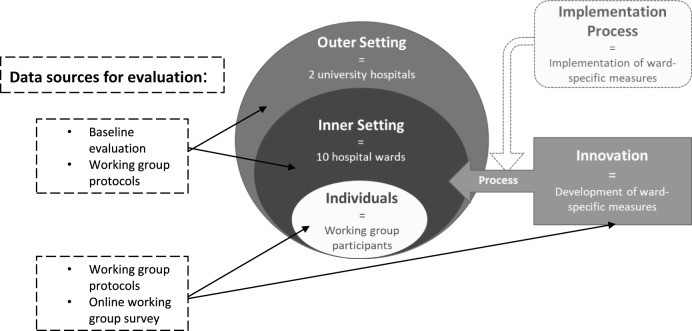
Operationalization of the major domains of the CFIR 2.0. Adapted from Damschroder et al. (2022). The updated consolidated framework for implementation research based on user feedback. Implementation Science, 17, 75. https://doi.Org/10.1186/s13012-022-01245-0. Image adapted by the center for implementation, © 2022. Version: V2024.01. https://thecenterforimplementation.com/toolbox/cfir.

We applied the *CFIR 2.0* to collect relevant factors for the evaluation of the WG process to ensure that all relevant factors that could influence the WG process are comprehensively addressed (Damschroder et al. 2022). It includes 5 major domains with respective constructs: outer setting, inner setting, characteristics of individuals involved, implementation process, and innovation. We adapted the constructs to our evaluation aims and data, e.g. by removing, redefining and/or adding new constructs. Figure 2 shows the operationalization of the major domains with data collection sources for evaluation and adapted constructs can be found in Supplemental material 1. We used data from the baseline evaluation and the WG protocols to contextualize how the development of measures is affected by the ward (= inner and outer setting). Collecting contextual data is crucial in implementation studies, as the effectiveness of interventions and their ability to reach all relevant target groups is critically influenced by the context in which they are implemented (Pfadenhauer et al. 2017). WG protocols and the concluding online WG survey were used to evaluate the implementation of the WG process (= innovation and individuals). For each ward, we created an Excel sheet containing the relevant data for the *CFIR 2.0* domains and constructs.

#### Baseline evaluation

Between September 2021 and April 2022, medical record analysis, focus groups, and a staff survey as well as interviews with informal caregivers were conducted on the respective wards. Details on methods used and results are published elsewhere (Kremeike et al. 2022; Meesters et al. 2025).

#### WG protocols

One research team member recorded details on each WG meeting, using a structured protocol. Therein, we documented meeting duration, number of participants, field notes on the procedure (e.g. discussions, selection of measures) and atmosphere. The research team debriefed each meeting to ensure the inclusion of all impressions in the protocol.

#### Online WG survey and qualitative feedback

To gather feedback on the WG process from the participants’ perspective, the research team conducted an online WG survey after the final meeting for each WG. The self-developed questionnaire consisted of 1 question regarding the profession, 16 question on satisfaction with the WG process (5-point-Likert scale) and 3 open-ended questions regarding motivation, insights and suggestions for improvement of the WG process. The survey link (LimeSurvey) was emailed to all WG participants (regardless of frequency of participations in meetings). The survey was open from 13 to 31 January 2023, with a reminder sent 2 weeks after the initial invitation. We also collected qualitative feedback in the final WG meeting by asking WG participants to give feedback regarding the following questions:

- Which things have worked well?
- What have we achieved/What have we learnt?
- What did not go well? (What was missing?)
- What would you have wished for/what do you still need?

### Data analysis

Qualitative data (staff focus groups, informal caregiver interviews, WG protocols) were analyzed by qualitative content analysis (Kuckartz 2012). We used a deductive-directed approach based on *CFIR 2.0*. WG protocols, the interviews, and focus group transcripts were uploaded into *MAXQDA 2022* and 4 researchers (S.M., S.K., V.M., C.H.) assigned relevant passages to the respective constructs of the adapted *CFIR 2.0*. The assignment was discussed constantly to clarify ambiguities and to foster a common understanding of domains and categories across all researchers.

Quantitative data (retrospective medical record analysis, staff and online WG survey) was analyzed descriptively (frequencies, percentages) using *IBM SPSS Statistics 28* (IBM Corp 2021; Kremeike et al. 2022) and *R Studio*. To inform the *CFIR 2.0* constructs, we selected suitable items and variables from the staff survey and the retrospective medical record analysis. Four-point Likert scales from the staff and WG surveys were condensed to 2 points (‘disagree’, ‘agree’), and 5-point scales were reduced to 3 points (‘disagree’, ‘neutral’, ‘agree’). Percentages were calculated for each of the response categories. Missing values were excluded from the analysis.

## Results

Firstly, we describe the context in which the WG process was implemented (outer and inner setting), based on baseline evaluation data (*n* = 400 medical records, *n* = 201 staff questionnaires, *n* = 10 staff focus groups, *n* = 12 interviews with informal caregivers) and *n* = 79 WG protocols. This is followed by the description of the WG process and its evaluation based on quantitative and qualitative feedback from the WG participants and WG protocols.

### Description of the context

- Outer setting: medical centers

The 10 participating wards are affiliated to 2 medical centers located in 2 large German cities, each city with over a million inhabitants. The medical centers comprise about 60 and 80 departments and institutes with 1500 and 1800 beds, respectively. The number of employees ranges between 11,000 and 14,900. Both hospitals offer a hospital palliative care support team alongside a palliative care ward. As the WGs began in February 2022, the process was impacted by staff shortages due to the COVID-19 pandemic and therefore increased workload and strain especially on ICUs. Additionally, one of the hospitals experienced a 11-week strike by nurses from April to July 2022.

1. Inner setting: hospital wards

The participating wards comprised 4 GWs (neurology; gastroenterology; internal medicine; radiotherapy) and 6 ICUs (internal [*n* = 2], surgical [*n* = 2], interdisciplinary [*n* = 2]). The mean number of deaths per year varied greatly, ranging from 11 to 200 (mean 2019–2022). To capture the inner setting, the authors assessed aspects related to communication and collaboration, on culture and resources already in place for the care of dying patients.

Communication and collaboration: The staff survey showed, that on 8 wards, the majority agreed that communication works well, while on 2 wards only 35.7% and 48.4% agreed, respectively (range 35.7–100%; mean 80.9%). Moreover, the staff only partly felt like being part of a multi-professional team (range 57.9–94.5%; mean 71.5%). On 4 wards, less than 90% of the staff agreed that there was a generally friendly climate (range 78.5–100%; mean 91.9%) and that team members treated each other respectfully (range 85.7–100%; mean 94.3%). The focus groups and WG protocols revealed that primarily nurses criticized an inadequate delivery of information and lack of exchange within the team. They wished for more inclusion in decision-making regarding changes of goal-of-care as they are closer to patients and their informal caregivers.

Culture: While staff on most wards reported that dying is accepted on their ward and that they are able to provide at least some dignity in dying, staff on 2 wards described a culture where dying is considered as a defeat. This culture impairs the care for dying patients from their perspective and leads to conflicts within the team.

Finally, the authors identified the main challenges that staff members perceive in caring for patients in the dying phase which could be assigned to 6 areas: (1) challenging and delayed decision making on change of goal-of-care, including involvement of patients and/or informal caregivers, (2) lack of time for and knowledge on care for dying patients, (3) inadequate exchange and information flow within the team and with other disciplines and professions, (4) challenging and inadequate information of patients and/or informal caregivers about the impending death, (5) lack of supporting structures for staff in case of strains, (6) lack of concepts for care of dying patients during weekends and nights. All wards already had individual resources to provide good care for the dying, e.g. farewell rooms. These were included in the planning of interventions.

### Description of the WG process

The WG process was structured with the same 3 steps for every WG. This standardized procedure was intended to ensure the comparability of the WGs, while also enabling a ward-tailored process. No predefined standards or measures were presented to the WGs at the beginning, in order to maintain the ED character of the process. Palliative care professionals were available throughout to support participants if needed and at least one member of the research team attended every meeting.

1. Presentation of ward-specific results from the baseline evaluation: In the first meeting, the research team provided ward-specific results from the baseline evaluation on resources, staff knowledge about care in the dying phase, the current situation, challenges of providing this care, and potential measures for improvement. The presentation of baseline results ensured that perspectives beyond those of the small number of WG participants were considered. It was also intended to help identify relevant areas for improvement.
2. Selection of prioritized topics: Using the baseline evaluation results, the WG identified areas needing improvement and prioritized them. To support the collecting process, methods such as mind mapping on a flipchart were used to visualize and cluster topics. Agreement on prioritized topics was reached through discussion or point vote.
3. Planning of ward-specific measures: In this final step, the WGs developed concrete measures based on the previously prioritized topics. To support the planning, we combined 2 approaches: First, the content-related planning process of the measures was aligned with the toolkit from the *AMBER Care Bundle* “Last Days of Life” (Koffman et al. 2019), which served as a structural guide. Second, the planning of the implementation of the measures was supported by an implementation model (Grol and Wensing 2020), which emphasizes systematic steps for translating ideas into practice. The WGs were encouraged to reflect on current practice and challenges related to the prioritized topics of care in the dying phase on their wards. Accordingly, they formulated *SMART* targets that are *specific, measurable, achievable, result oriented, and time scheduled* (Ogbeiwi 2017). Consequently, concrete ward-specific measures were derived. These included defined target groups, implementation steps, responsibilities, and timeframes. While the discussion was primarily driven by the participants, palliative care professionals were available to support the development of measures. In addition, the research team maintained a collection of evidence-informed measure examples based on a previous scoping review (Oubaid et al. 2025) to support the planning process when required, without superseding the participants own ideas.

WGs were established on all participating wards with 5 to 12 meetings throughout the year (*n* = 69 in total). The introductory meeting was planned for February and was realized for 5 WGs. The starts of the remaining WGs were delayed with the final WG starting in June. While the WG composition was multiprofessional, not every meeting was. For the number of participants, meetings and professions see Table 1. The WG composition varied over time: some wards (*n* = 7) maintained a permanent WG team, although not all participants attended every meeting. The other wards had a dynamic WG team with participants changing from meeting to meeting. Our standardized procedure to guide the WG process had to be tailored to meet the varying needs and requirements. For instance, the required meeting duration and structure varied between the WGs. Some WGs were more self-organized, directing the process, developing documents independently, and generating many ideas, while others needed more guidance and input from the research team.

**Table 1.** Overview of number of participants, professions, number of meetings of WGs

Hospital ward	Ø Number of participants	Profession*	Number of meetings
ICU 1	4	N, P	7
ICU 2	3	N, P, T	7
ICU 3	4	N, P	6
ICU 4	4	N, P, T	12
ICU 5	6	N, P, T	5
ICU 6	3	N, P, T, C	5
GW 1	5	N, P	8
GW 2	5	N, P, T, C	7
GW 3	5	N, P, C	6
GW 4	5	N, P, C	6

* N = nurse, P = physician, T = therapist (occupational, physical, and speech therapy), C = counselling.

The WG participants developed *n* = 34 measures during the WG process (see Table 2). The measures can be grouped thematically into the following topics:

- Informal caregivers; e.g. a flyer for the bereaved with support offers and information after the patients’ death.

**Table 2.** Overview of facilitators and barriers for the working group process at ward and WG level

Level	Facilitators	Barriers
Ward	High relevance of the topic dying and death on ward before initiation of the research study	Ward-specific dynamics/conflicts slowing down the process, e.g. strained team-intern relationships
	Ward staff is attuned to care need of patients and their informal caregivers in the dying phase	Institutional limits, e.g. non-possible electronical integration of documents
	Existing materials/structures to build on quickly	
Working group	Multiprofessional composition to integrate multiple perspectives	High fluctuation und declining participation of WG participants leading to cancellation and rescheduling of meetings
	Motivation and engagement of WG participants to e.g. gain knowledge and optimise care	Limited timely and personal resources/staff shortage impeding the overall WG process despite participants commitment and motivation
	Involvement of staff in managing positions (e.g. senior physicians) for faster task assignments and coordination	
	Involvement of ‘opinion leaders’ with strong opinions and high motivation impacting other WG participants	
	Combination of both small and easy to implement as well as larger and more intensive to implement measures to keep working group participants motivated
- Care in the dying phase; e.g. training for nursing staff regarding aroma therapy and oral care in the dying phase.
- Spatial setting; e.g. redesign of a farewell room.
- Team communication; e.g. a weekly multiprofessional palliative care team meeting.
- Supporting structures; e.g. overview of specialist palliative care structures and other support structures in hospitals for ward staff.

The measures aimed to facilitate communication processes within the team and with informal caregivers, to enhance knowledge and certainty in the care for patients in the dying phase, to establish supporting structures for the staff, and to improve the spatial concept of rooms for patients and their IC. The measures were tailored to the specific needs and structures of each ward. For example, 2 wards identified a need to improve team communication. One ward established a weekly multiprofessional team meeting to improve the overall internal communication and foster collaboration between nursing, medical, and therapeutic staff. Another ward introduced a monthly, interdisciplinary case discussion, primarily aiming to enhance communication between the ward team and other medical disciplines. These examples illustrate how the same general objective – improving communication – led to differently structured and solutions tailored to specific wards.

### Evaluation of the WG process

- Experiences and feedback from the perspective of the WG participants

In the following, the results of the online WG survey are presented as well as qualitative feedback that gathered in the final WG meeting. Of 78 invited, *n* = 44 (61.5%) completed the survey. The results will be reported according to organizational, communication/cooperation-related and outcome-related aspects. This is followed by the identification of barriers and facilitators of the WG process.

#### Organizational aspects

Most participants (70%) were satisfied with the organization of the WGs by the research team (e.g. scheduling, meeting rooms). Challenges arose in the scheduling of meetings due to shift schedules, particularly impacting nursing staff. Seventy-one percent of the participants felt that the time frame of the meetings was appropriate, with some wishing for continuation of meetings. Overall, 64% were satisfied with the WG process (realization, preparation, and follow-up work.)

#### Aspects related to communication/cooperation

Most participants (75%) rated the cooperation between the research team and the WG participants as satisfactory. Eighty percent felt they could express their opinion freely and 91% they could share their own thoughts and ideas. In the final meeting, participants highlighted the collaboration and cross-professional exchange, describing it as an ‘exchange at eye level’. Communication with and moderation by the research team was describe as professional and helpful.

However, participants wished for more involvement of managing positions, e.g. senior physicians, to accelerate development and coordination processes. The decreasing numbers of participants over time was perceived as negative for the process.

#### Aspects regarding the desired outcome

Participants were satisfied with results of the WGs (66%) and with the selection of measures on their ward (66%). The majority (77%) would be willing to participate in the WG again.

In the last meeting, participants generally found the selection of measures suitable, though one WG wished for more measures and the others wanted faster planning. One WG wished for more input regarding adequate care in the dying phase from palliative care experts during development, while others felt the process was adequate.

1. Barriers and facilitators of the WG process

We identified barriers and facilitators of the WG process based on the data of the WG protocols, which were grouped into 2 levels: ward and working group (see Table 2).

## Discussion

### Key findings

Research on the organization and conditions necessary for implementing ED innovations is limited. A recent scoping review found that only 12 out of 60 ED innovation studies included a robust evaluation (Cadeddu et al. 2023). Our study provided a comprehensive description and evaluation of an ED approach for tailoring measures to optimise hospital care in the dying phase. Baseline evaluation revealed considerable heterogeneity between the wards, highlighting the need for tailored interventions. We successfully established WGs on all participating wards and the WGs tailored 34 measures, demonstrating the feasibility and utility of the ED approach. While most participants viewed the WG process positively and would participate again, satisfaction with the results of the WGs and selected measures was mixed. Identified facilitators and barriers at both ward and WG levels provided valuable insights into the WG process.

### Feasibility and utility of the ED approach

The successful formation of WGs and participatory tailoring of measures confirm the feasibility of the ED approach. The baseline evaluation showed highly variable ward conditions, necessitating tailored interventions. Although all WGs consistently tailored measures within 5 thematic areas, the measures were individually designed to fit each ward’s unique requirements and structures. For instance, communication-focused measures ranged from interdisciplinary team meetings to efforts to improve documentation. Tailoring interventions and implementation strategies is indispensable for changes in complex healthcare settings (Baker et al. 2010; Geerligs et al. 2018; Wensing and Grol 2020). A systematic review on implementation processes for hospital-based interventions demonstrated the need to understand staff engagement and beliefs about the intervention and to generate strategies to address existing barriers (Baker et al. 2010; Geerligs et al. 2018). Our approach allowed tailoring from the beginning of both the measures and implementation strategies to the structural and staff requirements of the wards, ensuring flexibility for adaption. Another advantage was that the multiprofessional team appeared to benefit from working together in the WGs, which fostered communication and cooperation. Baseline findings indicated that staff members often felt only partially integrated into a multiprofessional team, and on some wards, only a minority agreed that communication works well. Bringing together the different professional groups in the WGs did not hinder open expression but instead fostered exchange at an equal level, promoting mutual understanding. Beyond tailoring measures, the WGs served as a platform to exchange views on patient care in the dying phase and to address internal conflicts or differing perspectives. A recent review on the implementation of ED innovations highlights that closer collaboration is an important benefit of these approaches on team level (Cadeddu et al. 2023).

Although the study demonstrated general feasibility of the ED approach, significant barriers to its implementation emerged. These barriers were rooted in well-known time and resource constraints within hospital settings. Despite high motivation and independently generated ideas, the integration of additional tasks into already demanding workloads led to fluctuations and a declining participation over time. This was despite the substantial organizational efforts undertaken by the research team to support the WG process. This aligns with existing research indicating that staff may perceive ED processes as extra work (Cadeddu et al. 2023). Additionally, only 66% of participants were satisfied with the results. Some measures, such as electronic documentation integration, could not be realized as desired by the WG. Others were not yet implemented at the end of the WG process due to unexpected delays. This reflects existing literature, pointing to the hospitals’ high level of complexity and lack of organizational resources as important barriers to successful implementation of interventions (Cadeddu et al. 2023; Fournier and Jobin 2018; Van Beers et al. 2022). Nevertheless, most participants endorsed the ED approach and would recommend it to other hospital wards.

### Key insights for transfer to other settings

Our detailed account of the WG process – including its structure, required resources, and processual adaptions – may serve as a practical orientation for other institutions aiming to apply similar ED approaches. As in most ED approaches (Cadeddu et al. 2023), the initiation of the WGs depended on the respective department directors’ approval, with staff leading the subsequent process. Therefore, it is crucial that directors, staff in leading positions and other staff members are motivated to optimise care in the dying phase. Some directors may underestimate the importance of palliative care and the need for improvement (Lind et al. 2017). To extend the process to other wards, ways to communicate the importance of palliative care on non-specialized wards and to motivate ward directors and staff to participate in such a process need to be elicited. In line with existing literature, staff in managing positions played a key role in successful tailoring the measures (Cadeddu et al. 2023; Van Beers et al. 2022), as their involvement streamlined coordination processes and task assignment. Recent reviews highlighted that effective change requires engagement across all hierarchical levels, with mid- and high-level managers functioning as role-models and bridging the gap between organizational directives and execution of measures (Cadeddu et al. 2023; Van Beers et al. 2022). This fosters a culture of co-creation and ownership of the measures at all hierarchical levels (Cadeddu et al. 2023; Van Beers et al. 2022). A well-balanced mixture of bottom-up and top-down approaches appears most effective, though further research is needed to determine the optimal ratio (Cadeddu et al. 2023).

For the ED approach to be effective, dedicated individuals must take responsibility for the process. In our study, the research team handled all organizational tasks. For broader implementation, hospitals must allocate personnel time and space to manage and oversee the process. External support may also be beneficial, as WG participants requested more external guidance and input. Staff members’ limited knowledge of potential measures may hinder the development of effective measures. For instance, the involvement of hospice services represents a valuable resource for improvement (Seaman et al. 2016), yet this was presumably not known and therefore not considered as an appropriate measure. In our study, specialized palliative care supported content-related questions but did not actively engage in tailoring measures. For future applications, intensifying the involvement of specialized palliative care could be beneficial, without limiting the autonomy of the WG participants.

### Strengths and limitations

One strength of the study is the use of the updated *CFIR* version, ensuring a structured evaluation of barriers and facilitators (Damschroder et al. 2022). Without frameworks, critical aspects may be overlooked, compromising validity and generalizability (Geerligs et al. 2018). We assessed the ward setting and challenges from staff perspectives, providing valuable practical insights. The inclusion of 10 GWs and ICUs across different departments of medical centers enhances the comparability and transferability of our results. Despite successful tailoring of measures, long-term sustainability remains uncertain. As planned, the research team withdrew after the WG process, preventing further involvement in the implementation of the measures. Further research examining the process after a longer period is necessary.

## Conclusion

The ED approach proved feasible and useful for tailoring of ward-specific measures aiming to optimise hospital care in the dying phase. Participants of the WGs tailored a wide range of measures and were generally satisfied with the process. Successful implementation requires individuals taking responsibility, engagement across all hierarchical levels, and external support. The optimal balance between bottom-up and top-down strategies remains unclear. The overview of identified measures provides a base to tailor own measures to optimise care in the dying phase on other wards. As part of the research project, the measures will be prepared and published more detailed for further use. When transferring the approach to other hospitals, the identified barriers and facilitators are an important guidance for effective implementation.

## Supporting information

10.1017/S1478951525100400.sm001 Meesters et al. supplementary material Meesters et al. supplementary material

## Supplementary material

The supplementary material for this article can be found at https://doi.org/10.1017/S1478951525100400.
