# Supplementary material for: Formative evaluation of an employee-driven approach to improve care in the dying phase in hospitals
Source: Palliat Support Care. 2025 Jul 18;23:e135. doi: 10.1017/S1478951525100400 (PMC13166520; doi:10.1017/S1478951525100400)
Supplement: Meesters et al. supplementary material [file S1478951525100400sup001.docx]

| 1. **DESCRIPTION OF THE SETTING** | |
| --- | --- |
| **1.1 University Medical Center** | |
| **Outer Setting Domain*** | |
| **Construct Name** | **Adapted Construct Description** |
| 1. Critical Incidents | Unexpected or major events that interfered tailoring of measures |
| 1. Specifics | Hospital-specific differences |
| *C.-G.* | *Not relevant.* |
| **1.2 Hospital ward** | |
| **Inner Setting Domain*** | |
| **Construct Name** | **Adapted Construct Description** |
| A. Structural Characteristics | Number of deaths, number of beds, patient characteristics (age, length of stay, diagnoses, communication skills) |
| 1. Physical Infrastructure | Space and equipment available on the ward (farewell room, consultation room, single room etc.) |
| 2. Information Technology Infrastructure | Exchange and documentation systems (paper based vs. electronic medical records, case reviews etc.) |
| 3. Work Infrastructure | Organization of responsibilities & tasks, staff structure, number of staff, staffing ratio |
| B. Relational Connections | Formal and informal relationships on the ward within the team/on the ward (therapists, surgeons) and outside (with palliative care specialist and other institutions) |
| C. Communications | Communication between ward staff |
| D. Culture | prevailing culture (more curative or palliative), norms, values |
| 1. Human Equality-Centeredness | Shared values, views and norms around the equal worth and dignity of every patient. |
| 2. Recipient-Centeredness |  |
| 3. Deliverer-Centeredness | Supporting and addressing the needs and well-being of ward staff |
| *4. Learning-Centeredness* | *Not relevant* |
| E. Tension for Change | Areas for improvement from the perspective of ward staff |
| F. Compatibility | Measures are tailored in the working groups. Procedures and processes are already considered during the process. In the context of this project, the compatibility of the measures is high because it is tailored and being adapted by ward staff. |
| G. Relative Priority | Description of recruitment of ward staff for the working groups |
| *H. Incentive Systems* | *Not applicable* |
| I. Mission Alignment | The measures and their implementation are in line with the objectives and obligations and purpose of the ward |
| J. Available Resources | Financial, spatial, material and timely resources for the study project |
| 1. Funding | Funding is available for wards |
| 2. Space | Spatial resources (e.g. farewell room) that can be used for the implementation of measures |
| 3. Materials & Equipment | Material resources (e.g. guidelines) that can be used for the implementation of measures |
| *K. Access to Knowledge & Information* | *Not relevant for pre-assessment* |
| **1.3 Ward staff/Working group members** | |
| **Individuals Domain*** | |
| **Roles Subdomain*** | |
| **Construct Name** | **Adapted Construct Description** |
| A. High-level Leaders | Ward staff with high authority and decision-making power (directors, senior physicians) |
| B. Mid-level Leaders | Ward staff with moderate authority and decision-making power  (physicians, team leaders) |
| C. Opinion Leaders | Opinion leaders on the wards/the working group |
| D. Implementation Facilitators | All persons with specialist knowledge who support the working group process |
| E. Implementation Leads | *Not relevant as constructs refer to implementation of measures* |
| F. Implementation Team Members |  |
| G. Other Implementation Support |  |
| H. Innovation Deliverers |  |
| I. Innovation Recipients |  |
| **Characteristics Subdomain*** | |
| **Construct Name** | **Adapted Construct Description** |
| A. Need | Strain of ward staff |
| B. Capability | *The individuals have interpersonal competencies, knowledge and skills to fulfill the role* |
| C. Opportunity |  |
| D. Motivation |  |
| 1. **REALISATION OF WORKING GROUP PROCESS** | |
| **Implementation Process Domain*** | |
| **Construct Name** | **Adapted Construct Description** |
| A. Teaming | Description of the working group meetings (number of meetings and participants, professions of the participants, description of the atmosphere) |
| B. Assessing Needs | Collecting information about needs, wishes and preferences for the measures and their implementation of patients and ward staff |
| 1. Innovation Deliverers |  |
| 2. Innovation Recipients |  |
| C. Assessing Context | Facilitators and barriers of the working group process |
| *D. Planning* | *Not relevant as information is collected in V. Implementation Process Domain* |
| *E. Tailoring Strategies* |  |
| *F. Engaging* |  |
| *1. Innovation Deliverers* |  |
| *2. Innovation Recipients* |  |
| *G. Doing* |  |
| H. Reflecting & Evaluating | Quantitative and qualitative feedback … |
| 1. Implementation | … on working group process |
| 2. Innovation | … on measures |
| I. Adapting | Measures will be tailored by working group |
| 1. **MEASURES** | |
| **Innovation Domain*** | |
| **Construct Name** | **Adapted Construct Description** |
| A. Innovation Source | Source of chosen measures |
| B. Innovation Evidence Base | Measures are evidence-informed |
| C. Innovation Relative Advantage | Advantages and benefits of chosen measures in contrast to current practice |
| D. Innovation Adaptability | Measures will be tailored to the needs and conditions of ward, see study protocol |
| E. Innovation Trialability | Measures can be tested before implementation |
| F. Innovation Complexity | Complexity of tailored measure |
| G. Innovation Design | Design of tailored measure |
| H. Innovation Cost | Cost of tailored measure and its’ implementation |
| **Implementation Process Domain*** | |
| **Construct Name** | **Adapted Construct Description** |
| A. Teaming | Description of the distribution of tasks and responsibilities for the implementation of the measure |
| B. Assessing Needs | Discussion of needs, wishes, preferences for the tailoring of measures |
| 1. Innovation Deliverers | See B. Assessing Needs |
| 2. Innovation Recipients | See B. Assessing Needs |
| C. Assessing Context | Facilitators and barriers of the tailored measure |
| D. Planning | Identification of roles and responsibilities, determination of steps and milestones, definition of goals and measures for successful tailoring and implementation |
| E. Tailoring Strategies | Tailoring strategy and theory for the individual measure |
| *F. Engaging* | Motivation of the participants to be part of the working group |
| 1. Innovation Deliverers | See F. Engaging |
| *2. Innovation Recipients* | *Not relevant as patients are not part of the working group* |
| G. Doing | Description of the implementation of the individual measures |
| H. Reflecting & Evaluating | Quantitative and qualitative feedback … |
| 1. Implementation | … on implementation outcomes |
| 2. Innovation | … on measure outcome |
| I. Adapting | Adaptation of the measure to the ward and its processes |

*Structure and names of domains according to Damschroder, L. J., Reardon, C. M., Widerquist, M. A. O., & Lowery, J. (2022). The updated Consolidated Framework for Implementation Research based on user feedback. *Implementation Science*, *17*(1), 75. <https://doi.org/10.1186/s13012-022-01245-0>
